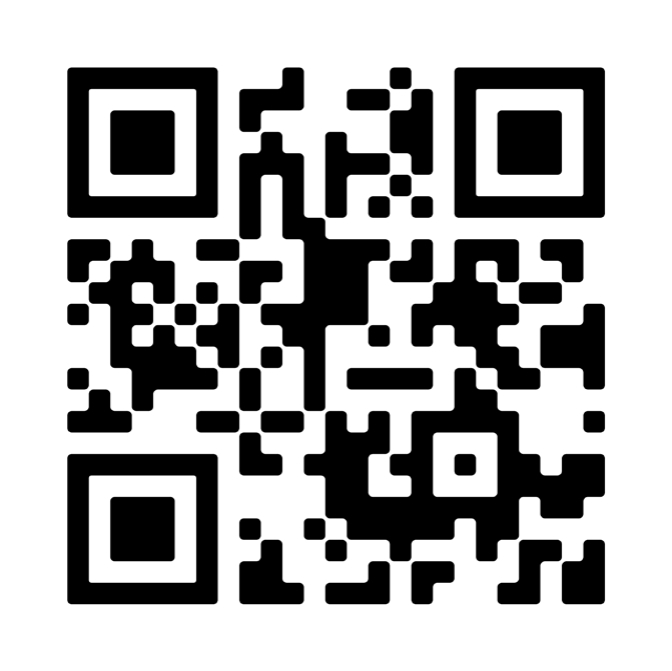# ICG mapping of postoperative lymphatic leakage in the groin: a video article and literature review

**DOI:** 10.52054/FVVO.16.3.033

**Published:** 2024-09-30

**Authors:** M Schubert, DO Bauerschlag, A Farrokh, N Maass, J Pape, I Alkatout

**Affiliations:** Department of Gynecology and reproductive medicine, University Hospital Jena, 07747 Jena, Germany; Department of Obstetrics and Gynecology, University Hospital of Schleswig Holstein, Campus Kiel, 24105 Kiel, Germany

**Keywords:** Inguinal lymphocele, lymphocele, lymphadenectomy, indocyanine green, vulvar cancer

## Abstract

**Background:**

Inguinofemoral lymphoceles are a common postoperative complication after inguinofemoral lymphadenectomy (LNE) and a challenge for patients as well as physicians. We report here our preliminary experience in the surgical management of a recurrent lymphocele using indocyanine green (ICG) detection, followed by robotic-assisted closure of the lymphatic leaks.

**Objectives:**

The aim of this article is to illustrate the surgical steps of ICG-assisted detection of inguinal lymphatic leaks and their surgical treatment by means of robot-assisted suturing. Furthermore, the feasibility of the approach will be evaluated.

**Materials and Methods:**

A 59-year-old woman with locally advanced squamous cell carcinoma of the vulva and previous conventional bilateral inguinofemoral LNE presented with symptomatic therapy-resistant lymphoceles in the groin. After a lengthy and frustrating course of standard therapy, she was offered the off-label option surgical treatment with ICG detection and subsequent robot-assisted ligation of the leaks, using the Da Vinci robotic system™.

**Main outcome measures:**

Perioperative data, specific aspects of the surgical approach specifics, objective and subjective outcomes of the new approach.

**Results:**

The procedure was performed as planned, with no intraoperative complications or device-related issues. The postoperative course was uneventful, and the patient developed no further lymphoceles.

**Conclusion:**

Visualisation of the leakage by ICG combined with minimally invasive robotic-assisted laparoscopy is a promising therapy option. The pictures and videos demonstrate our experience in regard of the safety, feasibility, and usefulness of this procedure. Further studies will be needed, to prove the absolute efficacy of the technique and express a general recommendation in regard of this approach for the treatment of inguinofemoral lymphoceles.

## Learning objective

Lymphoceles are a very common complication of LNE, particularly inguinofemoral LNE. The treatment of these lymphoceles is lengthy and frequently unsuccessful. Many patients require repeated transcutaneous punctures, medical therapies, or surgical treatment. Surgical options include lymphocele excision with ligation of the lymphatics or lymphatic venous shunts with primary closure with or without the insertion of a drain, or vacuum-assisted closure. A less invasive surgical therapy option is robot-assisted localisation of the leak by means of ICG with subsequent targeted closure, as described in this case report. This video shows the surgical steps of this approach using the Da Vinci system™.

## Introduction

**Figure 1:**
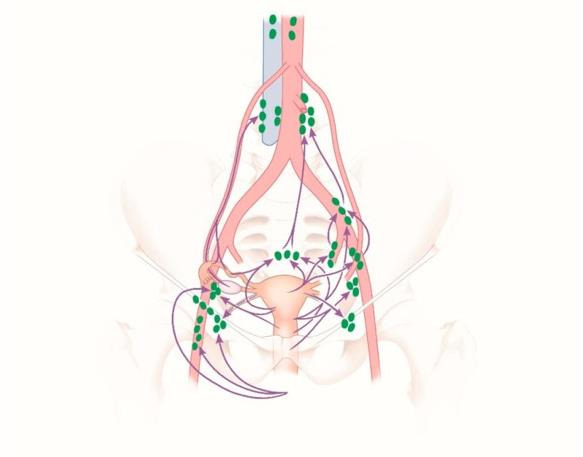
Lymphatic drainage of regional lymph nodes of the vulva and the female genital organs by Alkatout et al., 2015; Freytag et al., 2020. The individual lymph node groups are marked green; violet arrows indicate the lymphatic pathways.

With an incidence of 1.2 per 100,000 per year worldwide vulvar cancer is still very rare and accounts for a mere 4% of all gynaecological cancers (Olawaiye et al., 2021; World Health Organisation, 2023). Human papillomavirus (HPV) infection plays a major role in female cancers including vulvar, cervical, and vaginal lesions. Further risk factors are chronic vulvar dermatosis such as lichen sclerosis, smoking, and immunosuppression (Olawaiye et al., 2021, Oonk et al., 2023). Squamous cell carcinoma is the most common entity in vulvar cancer. Lymphatic drainage mainly occurs via the superficial inguinal lymph nodes. In contrast, tumours of the clitoris and the anterior labia minora may drain directly into the deep inguinal or internal iliac lymph nodes (Figure 1) (Alkatout et al., 2015).

Surgery by means of wide local excision (WLE) or hemi-/vulvectomy with or without sentinel lymph node excision (SLNE) or inguinofemoral/ pelvic LNE with the goal of R0 resection remains an important option in the treatment of vulvar cancer. Pelvic exenteration may be performed in large cancers or recurrent lesions after radiation therapy (Alkatout et al., 2015, Warmerdam et al., 2023, Fotiou et al., 1996, Oonk et al., 2023). Pelvic LNE and subsequent radiotherapy is advised in high-risk settings. In the adjuvant setting, radiotherapy in conjunction with chemotherapy is indicated in cases of positive margins without the possibility of resection or in the presence of risk factors such as lymphovascular space involvement (LVSI), perineural involvement (Pn1), or lymph node metastases (Nout et al., 2023, Oonk et al., 2023). Planning the treatment under computed tomography (CT), magnetic resonance imaging (MRI) or positron emission tomography (PET scan) guidance has facilitated intensity-modulated radiation therapy (Jhingran, 2022, Nout et al., 2023, Oonk et al., 2023).

## Patients and methods 

A 59-year-old woman with a locally advanced squamous cell carcinoma of the vulva, FIGO IIIc, cT2 G3 pN1 (by fine-needle biopsy), cM0, received a conventional inguinofemoral bilateral LNE, tumour resection with posterior hemivulvectomy, and partial colpectomy. The patient had a cervical carcinoma in 1999, which was treated by laparoscopic hysterectomy. Secondary conditions included arterial hypertension, hypercholesterolemia, and grade 3 obesity.

Primary radiochemotherapy as well as pelvic LNE were rejected by the patient. The final histology revealed FIGO IIIc, pT2 (25mm) pN2c (2/13, extracapsular spread in the left groin) G3 L1 V0 Pn1 pR1 (clinical R0), and HPV 16 positivity. The patient’s hospital stay was uneventful, and the inguinal drains were removed at a flow rate of < 30 ml/day.

The final recommendation was adjuvant radiochemotherapy with external radiation of the pelvis and groin, followed by high-dose-rate (HDR) brachytherapy.

During this period the patient presented with bilateral symptomatic, inguinofemoral lymphoceles 8 x 7 cm in size, which were repeatedly punctured by the transcutaneous ultrasound-guided technique. Since the treatment was not successful, we injected erythromycin (1g diluted in 20 ml sterile, pyrogen-free rinsing solution) into the areas of the lymphoceles. While the lymphocele on the right side resolved, a symptomatic lymphocele reappeared in the left groin. The various options were explained to the patient. She rejected further conservative measures. Owing to the size of the lymphocele, surgical revision with drainage was considered unpromising because it would not eliminate the actual cause.

## Results

After termination of transcutaneous radiotherapy of the groin, we performed surgery by ICG detection and subsequent robot-assisted ligation of the leaks. An 8-mm trocar was inserted 2 cm above the inguinal ligament. Distension of the wound cavity was achieved by administering saline. Under visualisation, two additional 8-mm working trocars were placed in the cranial pole of the large seroma cavity. The trocars were connected to the Da Vinci robot Xi systemTM (four arms, 30°, 8-mm fenestrated bipolar optic, large needle holder). The operation was performed under steady insufflation of lactated Ringer’s irrigation fluidTM. A total of 2 ml of ICG (25 mg in 10 ml saline) was then injected into the thigh area after disinfection. The lymphatic fistulae were visualised after three minutes by repeated flushing and release of water pressure and were then sutured with a 3.0 V-LocTM wound closure device. At the end of the operation, we observed no further leakage of ICG via the lymphatic channels. Fluid was aspirated and a 10-mm Redon drainTM was inserted with negative pressure. All instruments and trocars were then removed, and the incisions were closed with non- absorbable ProleneTM sutures.

**Figure 2:**
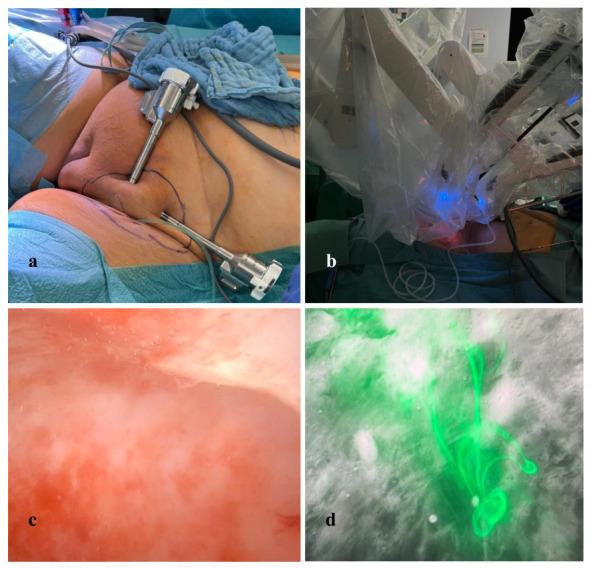
ICG mapping of the inguinofemoral lymphatic leakage followed by robot-assisted ligation of the leaks. a) positioning of the trocars, b) positioning of the Da Vinci robot, c) intraoperative visualisation of the lymphocele, d) ICG mapping of the lymphatic leakage.

A drain was placed at the end of the operation and removed after 4 days. The patient was discharged. The follow-up examination revealed a small quantity of fluid retention in the region of surgery, which was absorbed on its own. Figure 2 and the surgical video highlight the intraoperative leak visualised with ICG, and surgical treatment of the leakage.

## Discussion

Inguinofemoral LNE involves removal of lymph nodes along the inguinal ligament, within the proximal femoral triangle, as well as the deep portion of the cribriform fascia (Oonk et al., 2023). SLNE was established to attenuate the radical nature of surgery and thus significantly reduce complication rates by preserving lymphatics as well as surrounding vessels and nerves (Gaarenstroom et al., 2003, Van Der Zee et al., 2008, Rahm et al., 2022, Bekker and Meijer, 2008, Di Donna et al., 2023, Oonk et al., 2023). Thus, the major problem of lymphoceles is reduced but not eliminated by this approach.

Currently we have a variety of therapy options for lymphoceles: conservative management, recurrent transcutaneous punctures, sclerotherapy, injection of erythromycin, doxycycline or talc into the wound cavity, re-insertion of a drain, lymphocele excision with either ligation of the lymphatics or lymphatic venous shunts with primary closure with or without insertion of a drain, with or without a muscle flap, or with vacuum-assisted closure therapy (Hoffman et al., 1995, Khorram and Stern, 1993, Wallace et al., 2022, Sawhney et al., 1996). Despite the wide range of therapies, we lack guidelines for the treatment of lymphoceles (Gerken et al., 2020). Moreover, the techniques do not provide unequivocal success.

Therefore, the prevention of lymphoceles using a variety of options has gained increasing importance. The use of the PICO™ device, intraoperative drainage preservation of the superficial, deep fascia and saphenous vein, as well as multiple lymphatic venous anastomoses appear to reduce the risk of postoperative wound complications and lymphoceles (Asciutto et al., 2020, Wallace et al., 2022, Lawton et al., 2002, Boccardo et al., 2016, Saner et al., 2019).

A number of new options for the treatment of therapy-resistant lymphoceles are currently being tested. Dall et al. (Dall et al., 2020) achieved safe and promising results after the placement of silicone drains in the groin and the pouch of Douglas, which were then removed after three months under local anaesthesia.

A further novelty in inguinofemoral and pelvic LNE for vulvar carcinoma is video endoscopic inguinal lymphadenectomy (VEIL). Jain et al. (2017) and Liu et al. (2015) described robotic-assisted video endoscopic inguinal lymphadenectomy (R-VEIL) as a minimally invasive procedure with a low rate of surgical morbidity due to the preservation of the saphenous vein and its tributaries as well as the avoidance of inguinal skin defects and minimal reduction of blood supply to inguinal tissue. It remains to be seen whether VEIL will become a standard approach for LNE in vulvar carcinoma and whether the use of ICG will help to detect lymph nodes and lymphatics as well as prevent lymphatic leakage and the associated lymphoceles.

ICG is widely used in diagnostic investigation and surgery. Intraoperative ICG lymphography for confirming the patency of a lymphaticovenous anastomosis was described by Scaglioni et al. (2021) during the treatment of iatrogenic lymphoceles in the thigh.

Rebecca et al. (2019) performed a retrospective review of fifteen patients who underwent ICG lymphangiography during surgery for a lymphatic leak in the groin. The leak was identified successfully and treated with a local muscle flap. The number of patients treated by this technique so far are too small to provide sufficient evidence of its efficacy. A large prospective randomised multicentre trial will be needed before the technique can be recommended as a standard approach for the prevention of lymphoceles after LNE in the clinical setting. As of now, the procedure is a useful option for lymphoceles.

## Conclusion

Vulvar cancer continues to be a rarity among gynaecologic carcinomas although the number of patients, especially younger ones, is steadily increasing. Many attempts have been made in recent years to attenuate the mutilating nature of surgery for vulvar cancer. Yet, the iatrogenic complications of the treatment remain an unresolved problem. The use of ICG is safe and its applications are versatile. Moreover, ICG permits detection of lymph nodes as well as lymphatic channels and iatrogenic leakages, which can then be precisely treated surgically. Robot-assisted localisation of the leakage by means of ICG with subsequent targeted closure, as described in this case report, is a promising, safe, feasible and less invasive surgical option in selected cases.

Further studies will be needed to prove the efficacy of the technique and recommend its general use. The same is true of the potential preventive benefit of the procedure.

## Video scan (read QR)

https://vimeo.com/881983624/b73a44c3be?share=copy

**Figure:**